# A State-of-the-Art Review: Personalization of Tinnitus Sound Therapy

**DOI:** 10.3389/fpsyg.2017.01599

**Published:** 2017-09-20

**Authors:** Grant D. Searchfield, Mithila Durai, Tania Linford

**Affiliations:** Section of Audiology, Eisdell Moore Centre, The University of Auckland Auckland, New Zealand

**Keywords:** tinnitus, treatment, therapy, review, person-centered

## Abstract

**Background:** There are several established, and an increasing number of putative, therapies using sound to treat tinnitus. There appear to be few guidelines for sound therapy selection and application.

**Aim:** To review current approaches to personalizing sound therapy for tinnitus.

**Methods:** A “state-of-the-art” review (Grant and Booth, 2009) was undertaken to answer the question: how do current sound-based therapies for tinnitus adjust for tinnitus heterogeneity? Scopus, Google Scholar, Embase and PubMed were searched for the 10-year period 2006–2016. The search strategy used the following key words: “tinnitus” AND “sound” AND “therapy” AND “guidelines” OR “personalized” OR “customized” OR “individual” OR “questionnaire” OR “selection.” The results of the review were cataloged and organized into themes.

**Results:** In total 165 articles were reviewed in full, 83 contained sufficient details to contribute to answering the study question. The key themes identified were hearing compensation, pitched-match therapy, maskability, reaction to sound and psychosocial factors. Although many therapies mentioned customization, few could be classified as being personalized. Several psychoacoustic and questionnaire-based methods for assisting treatment selection were identified.

**Conclusions:** Assessment methods are available to assist clinicians to personalize sound-therapy and empower patients to be active in therapy decision-making. Most current therapies are modified using only one characteristic of the individual and/or their tinnitus.

## Introduction

State-of-the-art reviews are a specific form of review that focus on current issues and new perspectives, often in areas with a need of further research (Grant and Booth, 2009). The last 10 years has seen the emergence of many new tinnitus therapies using sound (Hoare et al., 2013a, 2014b). Although the heterogeneity of tinnitus is widely acknowledged by clinicians, many common sound-based tinnitus treatments are applied with limited assessment of individual differences (Hoare et al., 2014b). The ambiguity underlying tinnitus mechanisms and the rapid development of commercial interests in digital sound technology for tinnitus treatment have resulted in an increase in treatment options but few selection guidelines (Searchfield, 2016). There appears to be little information as to how to choose between treatments and how to apply them based on individual differences. In this state-of-the-art review we focus on how sound-based therapies are modified for individual characteristics and needs.

Research into the benefits of sound as a tinnitus therapy medium has not been systematic (Mckenna and Irwin, 2008; Hobson et al., 2012). There have been concerns as to whether the claims made regarding the effectiveness of sound therapy are correct (Mckenna and Irwin, 2008). We believe that some of the ambiguity surrounding sound therapy arises by applying the title “sound therapy” as a blanket term to the use of (any) sound that may have a positive effect on tinnitus. There are numerous mechanisms by which sounds could interfere with tinnitus (Norena, 2015). Tinnitus may be masked by sound interfering with encoding (e.g., energetic masking, Vernon, 1977) or pattern recognition (e.g., informational masking, Kidd et al., 1998, 2002). Sounds may desynchronize neural ensembles suspected in tinnitus generation (Eggermont and Tass, 2015). Sounds with positive emotional associations can affect mood and arousal (Handscomb, 2006; Tang et al., 2009). Hearing aids may mask tinnitus and have psychosocial benefits by improving communication (Shekhawat et al., 2013b; Searchfield, 2015). Long-term alleviation of tinnitus may occur through habituation (Jastreboff, 2000), inhibition (Teismann et al., 2011), gain reduction (Norena, 2015) or possible elevations in individual signal detection thresholds (Welch and Dawes, 2008). Attention, cognition and context of perception also appear to be important factors that manipulate long-term adaptation to tinnitus (Searchfield et al., 2012; Searchfield, 2014; Andersson et al., 2016).

Across the health sciences the personalized medicine movement has created a shift in focus from a “one size fits all” model to one that tailors diagnosis and treatment to the individual (Tutton, 2012; Schleidgen et al., 2013). At the same time health services are beginning to shift from evidence-based medicine to evidence-informed individualized care (Miles and Loughlin, 2011). Personalized medicine tends to incorporate traditional assessment methods, genotyping and genomic evaluation to explain and predict risk and treatment outcomes (Ginsburg and Willard, 2009). There is a search for biomarkers and endophenotypes in tinnitus (Sand et al., 2007). The current absence of clear genetic and blood-based biomarkers for tinnitus does not preclude personalization of treatments. The view of personalized medicine should be broadened away from just genetic markers (Anon, 2012). Tinnitus has both psychoacoustic (sound) (Tyler, 2000) and psychological (emotion, reaction) markers (Meikle et al., 2012). The context of tinnitus experience is also likely to affect its perception (Andersson et al., 2016) so that tinnitus is the result of psychosocial, psychoacoustic and individual psychological factors (Searchfield, 2014). In addition to modifying treatments based on assessment outcomes there are good arguments for applying principles of person-centered care to tinnitus therapy. In person-centered care patients are encouraged to be active participants in their treatment through the creation of a power-balanced, therapeutic relationship with their health professionals (Michie et al., 2003). Patients who participate in their own care report greater satisfaction, better adherence and health outcomes (Grenness et al., 2014). Research in the treatment of various health conditions such as chronic pain, balance disorders, and diabetes shows that self-efficacy beliefs also play an important role in treatment outcomes and management of the condition (Smith and Fagelson, 2011). Tinnitus self-efficacy is the confidence that individuals have in their capabilities to perform the treatment courses of action needed to manage their tinnitus successfully (Smith and Fagelson, 2011).

Person-centered needs-based care in rehabilitative audiology is not a new concept (Grenness et al., 2014). Most audiologists will be familiar with needs-based assessment for hearing aid selection (Dillon et al., 1987); similar principles could be applied in tinnitus therapy. Such an approach requires clinicians to take into account the needs of individual tinnitus patients, and provide custom-tailored therapeutic approaches. Our purpose in reviewing the literature was to identify the current state-of-the-art in personalizing sound-based therapies.

## Methods

A state-of-art review (Grant and Booth, 2009) was undertaken in December 2016 with cataloging of results in January and February 2017. All studies irrespective of quality were included as long as they addressed the topic and the research occurred in the last 10 years. There were no other inclusion/exclusion criteria. The research question for the current scoping review was: how do current sound-based therapies for tinnitus adjust for tinnitus heterogeneity? Sound-based therapies were defined as those that included the use of sound (either with or without counseling), but not psychological therapies used without sound or based on the scope of practice of psychologists (e.g., Cognitive Behavioral Therapy). From the included studies, all data were charted; themes and key issues were identified.

To identify relevant studies, the search was carried out using the databases Scopus, Google Scholar, Embase and PubMed for the 10-year period 2006–2016. The search strategy used the following key words: “tinnitus” AND “sound” AND “therapy” AND “guidelines” OR “personalized” OR “customized” OR “individual” OR “questionnaire” OR “selection.” The search on Google scholar was terminated when two full pages of consecutive results contained no entries of relevance to the study question. After initial consideration of title relevance to the study by one author (GDS), 199 articles were shortlisted. After reading the abstracts, 150 articles were read in full independently by two authors (GDS and MD). The reference lists of these 150 articles were searched for additional pertinent articles. This returned a further 15 articles for which the text was read in full, of those 165 articles for which full text was reviewed 83 studies described personalization (customization, individual adjustment) methods sufficiently to extract meaningful data. Two authors (GDS and MD) achieved a consensus on classification of content with the third author (TL) verifying categorization.

Studies were charted according to the method used to personalize therapy. The application of psychoacoustical and/or psychosocial assessment tools to treatment selection and management was cataloged along with the method used to customize the treatment for individual characteristics. A therapy might employ multiple types of customization and tools, in which case cross-referencing was used. In addition, evidence for person-centered care was recorded (including: getting to know patient or client as a person, sharing of power and responsibility, informed of treatment choice, accessibility and flexibility of service provider, coordination and integration, environment that is conducive to person-centered care).

## Results

Five themes were identified from the literature surveyed as categorical answers to the question: “how do current sound-based therapies for tinnitus adjust for tinnitus heterogeneity?” The themes were “hearing compensation” (adjustment to audiometry) “pitch matched” (adjustment using the predominant tinnitus pitch) “maskability” (adjustment to a desired level of masking) “reaction to sound” (selection based on the emotional or relaxing characteristics of sound) and “psychosocial factors” (use of psychological and/or environmental factors to select therapy). The therapy themes, the treatments using the approach, and the assessment used, are cataloged in Table 1.

**Table 1:** Therapy themes and references.

**Therapy theme**	**Treatments**	**Assessment**
**Hearing compensation**	**Hearing aids** Folmer and Carroll, 2006; Searchfield et al., 2010; McNeill et al., 2012; Peltier et al., 2012; Oz et al., 2013; Shekhawat et al., 2013b; Jalilvand et al., 2015; Searchfield, 2015; Sereda et al., 2016 **Threshold adjusted sound** Davis, 2006; Davis et al., 2007; Wazen et al., 2011; Peltier et al., 2012; Vanneste et al., 2013; Parra, 2015; Henin et al., 2016; Li et al., 2016 **Cochlear implant** Tyler et al., 2015	**Pure Tone Audiogram** Davis, 2006; Folmer and Carroll, 2006; Davis et al., 2007; Searchfield et al., 2010; Wazen et al., 2011; McNeill et al., 2012; Peltier et al., 2012; Oz et al., 2013; Shekhawat et al., 2013b; Vanneste et al., 2013; Jalilvand et al., 2015; Parra, 2015; Searchfield, 2015; Tyler et al., 2015; Henin et al., 2016; Li et al., 2016; Sereda et al., 2016
**Pitched-matched**	**Notched sound** Lugli et al., 2009; Courtenay et al., 2010; Teismann et al., 2011; Györi, 2016 **Band-pass noise** Serquera et al., 2015 **Tonal stimulation** Hanley and Davis, 2008; Reavis et al., 2010, 2012; De Ridder et al., 2015; Eggermont and Tass, 2015; Hauptmann et al., 2015, 2016; Hoare et al., 2015; Williams et al., 2015 **Auditory training** Herraiz et al., 2007; Hoare et al., 2010, 2014b; Jepsen et al., 2010; Roberts and Bosnyak, 2011; Spiegel et al., 2015; Wise et al., 2015, 2016 **Phase shifting** Herraiz et al., 2007; Vermeire et al., 2007; Choy et al., 2010; Meeus et al., 2010; Fioretti et al., 2011; Heijneman et al., 2012 **Hearing aids** Schaette et al., 2010; McNeill et al., 2012; Searchfield, 2016 **Replica tinnitus** Viirre, 2010; Drexler et al., 2016 **Emphasis tinnitus pitch** Bessman et al., 2009; Mahboubi et al., 2012	**Pitch matching** Herraiz et al., 2007; Vermeire et al., 2007; Hanley and Davis, 2008; Bessman et al., 2009; Lugli et al., 2009; Choy et al., 2010; Courtenay et al., 2010; Meeus et al., 2010; Reavis et al., 2010, 2012; Schaette et al., 2010; Fioretti et al., 2011; Roberts and Bosnyak, 2011; Teismann et al., 2011; Heijneman et al., 2012; Mahboubi et al., 2012; McNeill et al., 2012; Hoare et al., 2014a, 2015; Hutter et al., 2014; Eggermont and Tass, 2015; Hauptmann et al., 2015, 2016; Serquera et al., 2015; Spiegel et al., 2015; Williams et al., 2015; Györi, 2016 **Tinnitus Avatar** Viirre, 2010; Spiegel et al., 2015; Wise et al., 2015, 2016; Drexler et al., 2016
**Maskability**	**Partial masking** Tyler et al., 2007, 2012; Suzuki et al., 2016 **Mixing point masking** Henry et al., 2006; Huang et al., 2006; Jastreboff and Jastreboff, 2006; Jastreboff, 2007, 2011, 2015; Kim et al., 2014; Ostermann et al., 2016 **Hearing aids** McNeill et al., 2012; Searchfield, 2016 **Spatial** Oishi et al., 2013; Searchfield et al., 2016	**Patient report** Jastreboff and Jastreboff, 2006; Jastreboff, 2007, 2011, 2015; Tyler et al., 2007, 2012; McNeill et al., 2012; Oishi et al., 2013; Kim et al., 2014; Ostermann et al., 2016; Searchfield, 2016; Suzuki et al., 2016 **Calculation** Huang et al., 2006 **Localization** Searchfield et al., 2016
**Reaction to sound**	**Nature sounds / sound type** Handscomb, 2006; Ito et al., 2009; Piskosz, 2012; Herzfeld et al., 2014; Durai et al., 2015; Henry et al., 2015; Barozzi et al., 2016 **Music** Hann et al., 2008 Hann et al., 2008 **Notched music** Lugli et al., 2009; Courtenay et al., 2010; Teismann et al., 2011; Györi, 2016 **Filtered music** Davis, 2006; Davis et al., 2007; Hutter et al., 2014; Li et al., 2016 **Discomfort to sound** Bartnik and Skarzynski, 2006; Jastreboff and Jastreboff, 2006; Jastreboff, 2007, 2011, 2015; Durai et al., 2015	**Patient report** Davis, 2006; Handscomb, 2006; Davis et al., 2007; Hann et al., 2008; Ito et al., 2009; Lugli et al., 2009; Courtenay et al., 2010; Teismann et al., 2011; Piskosz, 2012; Herzfeld et al., 2014; Hutter et al., 2014; Durai et al., 2015; Henry et al., 2015; Barozzi et al., 2016; Györi, 2016; Li et al., 2016 **Categorization of report** Bartnik and Skarzynski, 2006; Jastreboff and Jastreboff, 2006; Jastreboff, 2007, 2011, 2015
**Psychosocial factors**	**Hearing aids** Searchfield, 2006; Hoare et al., 2012; Sereda et al., 2015 **Partial masking** Tyler et al., 2007; Anwar, 2013; Sereda et al., 2016 **Adaptation** Durai et al., 2015 **Mixing point masking** Mazurek et al., 2006; Herraiz et al., 2007; Ariizumi et al., 2010 **Selection** Tyler et al., 2006; Newman, 2008 #19; Newman et al., 2008; Tyler, 2012	**COSIT** Searchfield, 2006, 2015 **STOP** Newman et al., 2008 **TAQ** Tyler et al., 2006, 2007 **Demographics and clinical history** Mazurek et al., 2006; Herraiz et al., 2007; Hoare et al., 2012; Tyler, 2012; Sereda et al., 2015 **Motivation** Ariizumi et al., 2010 Ariizumi et al., 2010 **Personality** Tyler et al., 2006; Durai et al., 2015

*The references were categorized to the major theme of the research or review. There were occasions where references were cross-referenced to different themes or treatment categories. Assessment was broadly classified into categories; there was variation between studies in how specific features were measured (e.g., different forms of pitch matching). TAQ, Tinnitus Activities Questionnaire; STOP, Sound Therapy Option Profile; COSIT, Client Oriented Scale of Improvement in Tinnitus*.

### Hearing compensation

Treatments that modified their response on the basis of hearing sensitivity were hearing aids and sound stimulation compensating for reduced audibility (Table 1). Hearing aids were used to correct for loss of audibility of sounds that accompanied hearing loss. When the primary focus of hearing aid fitting was to improve hearing for speech an emphasis was placed on amplifying sound in a frequency specific manner to improve intelligibility (McNeill et al., 2012; Shekhawat et al., 2013b). When tinnitus was the primary focus a secondary goal for amplification was raising the audibility of environmental sounds (Shekhawat et al., 2013a). The basis for modification was the individuals hearing thresholds obtained using pure-tone audiometry. The amount of amplification was determined using a prescription based on the audiogram (Shekhawat et al., 2013a). Hearing aids were considered to reduce hearing handicap, reduce the levels of attention paid to tinnitus, and compensate for deafferentation, and possibly improve cognition (Searchfield, 2006, 2015; Shekhawat et al., 2013b; Sereda et al., 2015; Zarenoe et al., 2017). McNeill et al. (2012) identified those most likely to achieve benefit from hearing aids as having good low frequency hearing and tinnitus pitch within the amplification range of the hearing aids (McNeill et al., 2012). Jalilvand et al. (2015) suggested that hearing aids might be more successful in management of tinnitus from blast injuries than sound generators (Jalilvand et al., 2015). Frequency lowering processing was suggested as an alternative strategy to conventional amplification (Peltier et al., 2012). Several studies suggested sound therapy device selection based on the audiogram (from hearing aids, combination instruments and cochlear implants) (Folmer and Carroll, 2006; Mazurek et al., 2006; Tyler et al., 2015; Searchfield, 2016). Searchfield (2016) recommended normal hearing would be fitted with sound generators; high frequency hearing loss with hearing aids; hearing loss encompassing low frequencies with combination instruments; severe-profound hearing loss with cochlear implants.

Hearing loss will affect the perception of therapeutic sounds in addition to reducing the audibility of speech and environmental sounds. To address this several sound therapies adjusted the spectrum of music (Davis et al., 2007; Wazen et al., 2011; Peltier et al., 2012; Vanneste et al., 2013; Henin et al., 2016; Li et al., 2016), fractal tones (Herzfeld et al., 2014) or noise (Uriz et al., 2013) for the presence of hearing loss. The intent from threshold-adjusted sounds was to make the sound audible across a broad frequency range rather than stimulating the frequencies with best thresholds (Davis, 2006; Távora-Vieira et al., 2011). In some cases too severe a hearing loss was an exclusion factor for therapy (Davis et al., 2007). One study suggested that adjusting music levels for hearing threshold was of no benefit to tinnitus suppression (Vanneste et al., 2013).

### Pitch-based therapy

Several therapies used the pitch or spectrum of tinnitus as the basis for stimulation. Sound stimuli were individualized to span a frequency range centered on the dominant tinnitus pitch (Table 1). The sounds used and intended mechanisms of effect varied greatly: some therapies attempt to change the synchronized firing of neural assemblies near tinnitus pitch using tonal stimulation (Hanley and Davis, 2008; Reavis et al., 2010, 2012; Eggermont and Tass, 2015; Hauptmann et al., 2015, 2016; Hoare et al., 2015; Williams et al., 2015); others changed the phase of sounds presented at tinnitus pitch (Herraiz et al., 2007; Vermeire et al., 2007; Choy et al., 2010; Meeus et al., 2010; Fioretti et al., 2011; Heijneman et al., 2012) and another paired tonal stimulation with vagus nerve stimulation (De Ridder et al., 2015). Tinnitus pitch was used as the basis for selecting band-pass noise (Serquera et al., 2015) notched music (Courtenay et al., 2010; Teismann et al., 2011; Györi, 2016) or noise (Lugli et al., 2009) for lateral inhibition (Courtenay et al., 2010) and one method provided extra stimulation at tinnitus pitch (Mahboubi et al., 2012) while another used tinnitus pitch-matched sound embedded in nature sounds as a therapy (Bessman et al., 2009). Another form of pitch-based therapy employed participants undertaking active training tasks in discrimination (Herraiz et al., 2007; Roberts and Bosnyak, 2011; Hoare et al., 2013b, 2014b; Wise et al., 2016) or categorization tasks (Jepsen et al., 2010). The intended mechanism of effect for these training tasks is reorganization of tonotopic maps, but their main effect may be in modifying attention to tinnitus (Hoare et al., 2010). Several studies used more complex replicas (avatars) of tinnitus for passive stimulation (Viirre, 2010) and stimulation only while asleep (Pedemonte et al., 2010). Auditory training in a game format used the individual's tinnitus avatar as a distractor (Wise et al., 2016).

The variability in tinnitus pitch matching is a critical concern issue for pitch-based treatments (Hoare et al., 2014a; Serquera et al., 2015). Variability in pitch match that is more than one octave between consecutive sessions may preclude some therapies (Hoare et al., 2014a). Pitch matching is not considered very useful in methods based on counseling and broad noise therapy (Baguley et al., 2013). However, tinnitus pitch within the effective range of sound therapy device may be a prognostic factor for treatment success (Schaette et al., 2010; McNeill et al., 2012; Searchfield, 2016). Momentary analysis may have a role in guiding treatments in which the feature measured (e.g., pitch) guides treatment sound selection. Incorporating such assessments into daily routine in a non-threatening manner or even game (Wise et al., 2016) may mitigate the potential negative effects of momentary analysis in priming individuals to focus on their tinnitus.

### Maskability

We used a psychoacoustic definition of masking: when the perception of tinnitus is affected by the presence of another sound. The level of sound used in theory has been one of the more contentious issues in audiology-based tinnitus therapy (Jastreboff, 2007; Tyler et al., 2012). Masking can be used to totally or partially reduce the audibility of tinnitus by covering it with another sound. Tinnitus Retraining Therapy (TRT) advocates a masking level in which the sound mixes with, but does not cover, the tinnitus (Henry et al., 2006; Huang et al., 2006; Jastreboff and Jastreboff, 2006; Jastreboff, 2007, 2011, 2015; Kim et al., 2014; Ostermann et al., 2016) while others suggest use of the minimum level resulting in relief (Tyler et al., 2007, 2012; Suzuki et al., 2016). Most therapies set their target level based on patients' reports of tinnitus audibility and sound being comfortable, although Huang et al. (2006) reported that the mixing level could be predicted on the basis of the MML (Huang et al., 2006). Kim et al. (2014) reported TRT with broadband noise to have a higher success rate than mixed or narrowband noise (Kim et al., 2014). TRT and masking is typically practiced using sound presentation to both ears; Oishi et al. suggested that monaural presentation can be successful (Oishi et al., 2013) and Searchfield et al. (2016) showed the potential for binaural sound presentation using interaural cues and Head Related Transfer Function to achieve spatial as well as spectral masking.

### Reaction to sound

In order for sound therapy to be effective it must be comfortable to the user. Under this theme we cataloged therapies that considered individual's sensitivity to, or, reaction to sounds. A key factor in allocating participants to the treatment categories used in TRT was known discomfort to sound (Jastreboff and Jastreboff, 2006; Jastreboff, 2007, 2011, 2015). Personality may be a predictive factor in determining if a participant is sound responsive or sound sensitive (Durai et al., 2015). A positive individual response to therapeutic sounds will increase the individual's ability to achieve treatment goals as well as compliance to treatment. The strong emotional response to music has seen its use as a therapeutic tool (Hann et al., 2008). Music has been adopted as the sound manipulated in the Neuromonics Tinnitus Treatment (Davis et al., 2007; Li et al., 2016) and notched music therapies (Lugli et al., 2009; Courtenay et al., 2010; Teismann et al., 2011; Györi, 2016). Fractal tones also have music-like relaxation properties (Herzfeld et al., 2014). Advances in hearing aid technology allow relaxing music or nature sounds to be directly streamed from a patient's smart phone to their hearing aids (Piskosz, 2012). Studies to date suggest that modulated sounds (Henry et al., 2015) or nature sounds (Barozzi et al., 2016) achieve tinnitus benefits similar to broadband noise stimulation.

### Psychosocial

We defined psychosocial factors in sound therapy as social moderators and individual thoughts and behaviors that determine the treatment approach. In an evaluation of tinnitus management in NHS audiology departments in the UK by Hoare et al. (2012) identified a wide range of factors that influenced clinicians management strategies (in order of high-low reporting): level of hearing loss, evidence of stress or anxiety, state of mind, severity, willingness to try treatment, sleep disturbance, health, understanding, lifestyle preferences, coping ability, hyperacusis, age, and depression. Although these results were not described in terms of sound-therapy specific decision-making, three of the top four treatments reported were sound-based or could use sound (hearing aids, sound generator, habituation). In a similar population psychosocial factors were used in selecting hearing aids for tinnitus and mild hearing loss (Sereda et al., 2015). Skepticism, length of treatment and attitude can influence treatment success (Herraiz et al., 2007). Willingness to pay may affect client decision-making (Tyler, 2012). Older patients and tinnitus of longer duration may benefit less from sound therapies (Mazurek et al., 2006; Anwar, 2013). Questionnaires have been used in several person-centered tinnitus therapies to guide treatment (Table 2). In Tinnitus Activities Treatment (TAT), the Iowa Tinnitus Activities Questionnaire is recommended to identify patient's needs and treatment priorities (Tyler et al., 2007). Searchfield (2006) advocated the use of a tinnitus version of the Client Orientated Scale of Improvement (COSIT) to identify and set goals for treatment. Newman et al. (2008) used the Sound Therapy Option Profile (STOP) to assist in therapy selection and understanding patient attitudes to different treatments and the Self-Efficacy for Tinnitus Management Questionnaire (SETMQ) can be used to assess patient confidence in using different treatments (Smith and Fagelson, 2011; Fagelson and Smith, 2016).

**Table 2:** Examples of tinnitus assessments that can be chosen to help guide sound therapy selection.

**Assessment tool**	**Suggested use**
TFI (Meikle et al., 2012) TPFQ (Tyler et al., 2014)	Intake and outcome questionnaires developed to be sensitive to treatment effects. Determine effect of tinnitus on individual and areas of life most affected by tinnitus. Assist in priority setting
Audiometry (Sereda et al., 2011)	Identify degree of hearing loss accompanying tinnitus, as basis for modifying audibility of sound therapy
HHI (A or E) (Zarenoe et al., 2017)	Assists in determining if hearing aids should be trialed based on effect of hearing
Psychoacoustic matching (Henry et al., 2013; Hoare et al., 2014a)	Identify tinnitus characteristics on which to base sound stimulation. Essential for pitch-based therapies, helpful in predicting hearing aid success
HADS (Zigmond and Snaith, 1983)	Measure of anxiety and depression, assists in identifying need for referral and focus of therapy (e.g., CBT vs. sound therapies)
MPQ (Tellegen, 1982; Durai and Searchfield, 2016)	Personality typing helps identify sound sensitive patients and tendency for chronic tinnitus
NIH Cognitive Toolbox (Heaton et al., 2014)	Assess cognition (attention, memory) have influence therapy selection and settings (e.g., sow processing for hearing aids)
TAQ (Tyler et al., 2006, 2007)	Identify activities requiring therapy focus
STOP (Newman et al., 2008)	Assists selection of sound therapy types
SETMQ (Smith and Fagelson, 2011)	Identify areas where patients are struggling to manage tinnitus
COSIT (Searchfield, 2006)	Identify and prioritize individuals needs and goals

*TFI, Tinnitus Functional index; TPFQ, Tinnitus Primary Function Questionnaire; HHI (A or E), Hearing Handicap Inventory (Adults or Elderly); HADS, Hearing Anxiety and Depression Scale; MPQ, Multidimensional Personality Questionnaire; TAQ, Tinnitus Activities Questionnaire; STOP, Sound Therapy Option Profile; SETMQ, Self-Efficacy for Tinnitus Management Questionnaire; COSIT, Client Oriented Scale of Improvement in Tinnitus*.

## Discussion

Tinnitus is a heterogeneous disorder: tinnitus sound can differ between individuals, it can result from many different types of injury and its effect can vary from a minor annoyance to catastrophic impact on daily life (Stouffer and Tyler, 1990).

The review of the literature identified five sound therapy themes. Some treatments were included across themes. The majority of studies considered tailored or customized therapy to be selection of treatment sound on the basis of either audiometric threshold or tinnitus pitch. There were approaches that used dimensions of tinnitus severity, sound sensitivity and hearing to categorize or subtype groups of sufferers (Jastreboff, 2011). Another approach was a hierarchy or stepped care model in which individually tailored treatments were used if less resource intensive methods were unsuccessful (Myers et al., 2014). Stepped care has been implemented in environments where universal individually focused therapy would be economically unsustainable (Department of Health, 2009; Myers et al., 2014). Unlike single characteristic therapies whole-person approaches involved patients in decision making as to which of several approaches are best suited to them (Tyler et al., 2007; Newman et al., 2008). We believe that a contributing factor to the inconsistent benefit reported with sound therapies (Mckenna and Irwin, 2008; Hobson et al., 2012) is application of a single title “sound therapy” to a very heterogeneous collection of different sound-based approaches. In addition individual needs and reaction to therapy sounds differ (Durai and Searchfield, 2017) we ascribe to the philosophy that individuals are most likely to manage their tinnitus better when the treatment plan is tailored to their needs (Fisher and Boswell, 2016).

### Planning individual tinnitus care

The review did not identify any comprehensive guidelines for optimal sound therapy selection. We believe that many of the sound therapies identified could be effective when selected for the right patients at the right time and appropriate context. To do this we suggest careful assessment and then use of an individual care plan. In this review we deliberately focus on sound therapy, but we strongly believe any treatment plan should consider counseling (Tyler et al., 2007; Searchfield et al., 2011), and referral for psychological therapies such as Cognitive Behavioral Therapy (Martinez-Devesa et al., 2006) when appropriate. By determining individual needs and priorities, alongside assessment measures such as pure-tone audiometry and pitch matching, a plan can be developed that we believe reduces the risk for ineffective treatment. An effective individual care plan may also reduce the time required for treatment, reducing stress, anxiety and loss of hope for the sufferer. We believe those factors of the individual's complaint that are likely to be driving other symptoms should be addressed by therapy first (Fisher and Boswell, 2016).

Based on the review, along with our clinical experience, we suggest that the individual care plan use various clinical assessment methods. When patients first attend the clinic their clinical history, a thorough hearing assessment and tinnitus matching should be undertaken (Langguth et al., 2007). A questionnaire assessing aspects of tinnitus effects on quality of life should be used to provide an overview of tinnitus impact, and serve as a baseline for future assessments of outcomes. The Tinnitus Functional Index (Meikle et al., 2012) and Tinnitus Primary Function Questionnaire (Tyler et al., 2014) are two recent questionnaires developed for this purpose. Based on a clinical history an evaluation of anxiety and depression (e.g., the Hospital Anxiety and Depression Scale, Andersson et al., 2003) or cognition [e.g., National Institutes for Health (NIH) Toolbox Cognition Battery, Heaton et al., 2014] may be important. The comorbidity of anxiety and depression with tinnitus is well known (Andersson et al., 2003). The Hospital Anxiety and Depression Scale (HADS) (Zigmond and Snaith, 1983) or similar questionnaire scores can assist decision-making as to the necessity and priorities for psychological therapies. Tinnitus negatively impacts on cognition (Zarenoe et al., 2017). We do not know yet if differences in cognition should influence selection of sound therapy type, however research suggests that slow-acting hearing aid processing strategies may lead to better hearing when users memory is impaired (Lunner et al., 2009). We also recommend the assessment of personality. The use of personality questionnaires such as the Multidimensional Personality Questionnaire (MPQ) subscales (Tellegen, 1982) in clinics may be useful in identifying at-risk individuals for distressing tinnitus. Four key “maladaptive” personality traits are suspected in playing a role in diverting attention and processing resources toward tinnitus and which may subsequently act to prevent adaptation. These include higher levels of stress reaction, lower social closeness, lower self-control and higher alienation (Durai and Searchfield, 2016). If, for example, an individual has high stress reactions and low self-control and reacts negatively to sound psychological-based interventions may be needed before sound therapy.

Hoare et al. (2014b) recommended that clinicians be guided by the patient's point of care, patient motivation and expectations of sound therapy. The acceptability of the intervention both in terms of the sound stimuli to be used and whether patients are willing to use sound extensively or intermittently is important (Hoare et al., 2014b). A step in this direction is counseling patients about the therapies that are available. Information should be provided about the basis of the treatment, evidence for effectiveness, speed of effects, and costs. Aazh et al. (2009) suggest a poster format for this pre-consultation information; clinics websites and marketing material also can provide useful appointment scene setting. A tinnitus needs assessment can assist in the shared decision making process. The Hearing and Tinnitus clinic at the University of Auckland has used the COSIT as a decision making and goal setting tool for over a decade (Searchfield, 2006). The COSIT is a modification of the COSI, a tool frequently used in needs assessment for hearing problems (Dillon et al., 1987). Other questionnaires that may assist needs assessment include the TAQ (Tyler et al., 2007), STOP (Newman et al., 2008) and SETMQ (Smith and Fagelson, 2011). The TAQ determines the areas in which tinnitus creates problems (emotion, sleep, communication and/or concentration). The TAQ can highlight areas of life in which tinnitus is having the most debilitating effect, which can then be used to focus or tailor treatment (Tyler et al., 2007). The STOP is an 11-item tool that takes into account motivation, acceptance, expectations and willingness to use sound therapy devices (Newman et al., 2008). The SETMQ is a 40-item measure that quantifies the patient's confidence in managing tinnitus in five areas: (1) routine management, (2) emotional response, (3) internal thoughts and interaction with others, (4) tinnitus concepts, and (5) use of assistive devices such as hearing aids and maskers (Smith and Fagelson, 2011).

The relative effectiveness of these questionnaires in informing successful treatment has to be determined. However the usefulness and time savings achieved by use of these questionnaires should not be underestimated, as they can be completed by the patient prior to their appointment and can be assessed by the clinician prior to meeting the patient; saving time during the appointment. There is overlap in the questions asked by the questionnaires so not all need be used. It is important that clinicians choose those questionnaires best suited to the treatments they offer and their health care setting and patient population. We suggest that many audiologists will find the open-ended format of the COSIT familiar, and may wish to combine with one or more closed question formats (TAQ, STOP, SETMQ). The outcome of appropriately selected assessments can result in an efficient tinnitus clinic. Unnecessary or inappropriate treatments can be avoided, reducing the risk that patients become disillusioned with the clinician's methods. With patient “buy-in,” motivation and compliance to treatments should be high. Understanding and choosing the treatments to use immediately, and as the impact of tinnitus changes, may be empowering to the patient.

### Advancing sound therapy and recognizing its limitations

The literature review highlighted the diverse basis and application of sound therapy. Researchers and publications need to be clear on what aspect or type of sound therapy is being used. Mckenna and Irwin (2008) wrote a useful critique of sound therapy with the provocative title “Sound therapy: sacred cow or idol worship?” Mckenna and Irwin's (2008) main arguments were that the mechanisms of sound therapy were not necessarily those claimed, effects may be cognitive or psychological rather than purely auditory, and benefits were modest, if any, above counseling alone. Sound therapy is potentially confusing, given the numerous approaches and various potential mechanisms of effect. While more evidence for sound therapies is becoming available there is still a need to prove benefits. The individualized sound therapy approach also needs to be validated relative to single therapy protocols. In order to provide this evidence we may a need to move away from the dominant nomothetic research approach to an idiographic method that embraces individual variance (Fisher and Boswell, 2016). Group comparisons are limited in their ability to identify effective sound therapies when there is heterogeneity. Sound therapy is, in general, a slow-acting therapy that requires long-term use of some form of sound delivery device; so longitudinal data is needed. Research also needs to continue to investigate whether auditory-based therapies can be enhanced, or sped up, by combining with non-invasive brain (Shekhawat et al., 2014; De Ridder et al., 2015) or multisensory (Spiegel et al., 2015) stimulation.

## Conclusions

The basis of sound therapy is the belief that increasing extrinsic sound driven activity of the auditory system reduces tinnitus. This does not mean sound therapy is uniform in its application; instead it covers many dimensions and presumed mechanisms of effect. Current commentary on sound therapy fails to fully recognize this heterogeneity in application. At the same time few sound therapies can truly be considered personalized to make the most of their purported mechanisms. Much of the literature surveyed used the terms “customized” or “tailored” in terms of a single dimension rather than viewing tinnitus as a complex combination of dimensions. Tools exist for personalizing and planning treatments, they should be integrated into patient care, and their usefulness tested.

## Author contributions

GS undertook the initial database search, cataloging, and prepared the manuscript and revisions. MD was involved in manuscript preparation and cataloging of results. TL reviewed the cataloging and contributed to the manuscript with a focus on clinical application.

### Conflict of interest statement

GS is the scientific director of the University of Auckland Hearing and Tinnitus Clinic and Tinnitus Tunes, an online Tinnitus Therapy resource. The other authors declare that the research was conducted in the absence of any commercial or financial relationships that could be construed as a potential conflict of interest.
